# A Case of Myocarditis and Near-Lethal Arrhythmia Associated With Interleukin-2 Therapy

**DOI:** 10.1177/2324709617749622

**Published:** 2018-01-24

**Authors:** Stephanie Wu, Anna Sarcon, Khuyen Do, Jerold Shinbane, Rahul Doshi, Helga Van Herle

**Affiliations:** 1 University of Southern California, Los Angeles, CA, USA

**Keywords:** ventricular tachycardia, myocarditis, IL-2 therapy

## Abstract

We present a case of a 48-year-old female who developed myocarditis and near fatal arrhythmias during high dose Il-2 therapy for metastatic renal cancer. On day 5 of therapy, the patient developed sudden onset chest pain, elevated cardiac enzymes and ST segment changes on EKG. Coronary angiogram was normal, however echocardiogram showed reduced ejection fraction and hemodynamic measurements showed elevated bilateral elevated filling pressures. The patient then developed episodes of recurrent ventricular arrhythmia, precipitated by bradycardia and PVC, requiring defibrillation and temporary pacemaker placement. Endomycardial biopsy was nonspecific showing fibrosis with subsequent cardiac MRI showed evidence of myocardial edema, consistent with Il-2 induced myocarditis in the setting of no prior cardiac history. After the discontinuation of Il-2 therapy, the patient displayed clinical improvement as well as improved ejection fraction. This case brings attention to the cardiac toxicities associated with high dose Il-2 therapy including potentially lethal arrhythmias and highlights the importance of careful cardiac screening prior to initiation of treatment.

## Introduction

High-dose interleukin-2 (IL-2) is a therapeutic option for patients with metastatic renal cell carcinoma and has been associated with clinical response in up to 20% of these patients. IL-2 therapy can be associated with many side effects including hypotension, oliguria, and diarrhea, which are thought to be because of capillary leak syndrome. Cardiac side effects are common with IL-2 therapy and range from sinus and supraventricular tachycardia to cardiomyopathy and myocarditis; however, most side effects appear to be reversible with discontinuation of therapy.^1,2^ In this article, we report a case of a patient who developed myocarditis and near-lethal arrhythmia after receiving high-dose IL-2 therapy.

## Case Presentation

**Figure 1.**
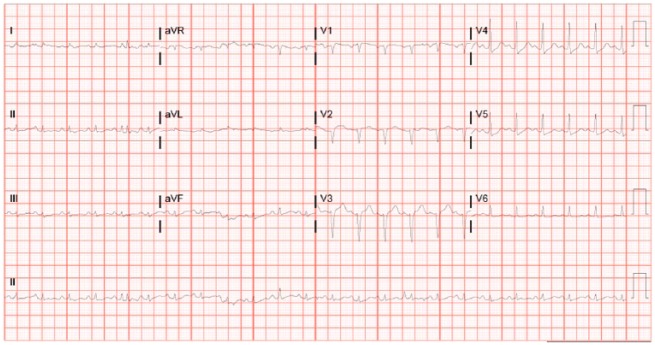
Sinus tachycardia with low voltage in limb leads and anterolateral ST depression.

**Figure 2.**
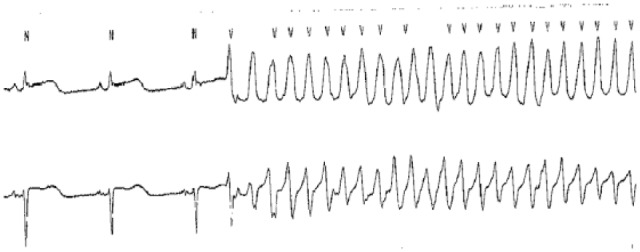
Rhythm strip showing sinus bradycardia with premature ventricular contraction occurred on antecedent T-wave leading to polymorphic ventricular tachycardia.

**Figure 3.**
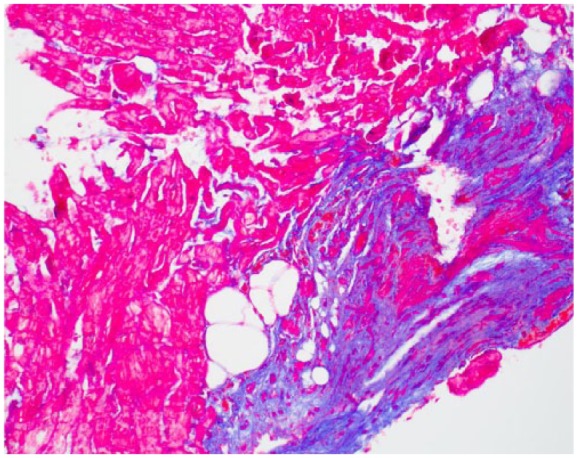
Myocardial tissue with trichrome stain showing focal fibrosis.

**Figure 4.**
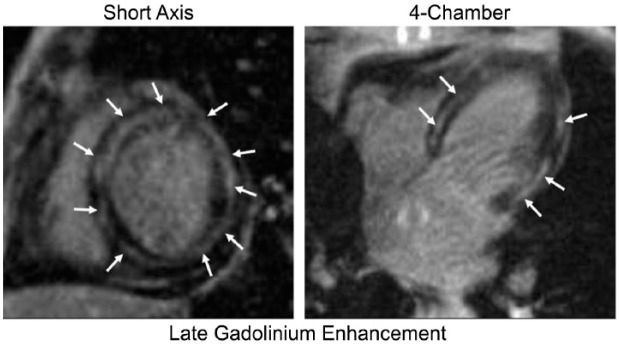
Cardiac magnetic resonance imaging showing enlarged left ventricle with wall thinning. Patchy mid-wall and scattered areas of subepicardial delayed gadolinium enhancement (arrows) involving all segments of the left ventricle.

**Figure 5.**
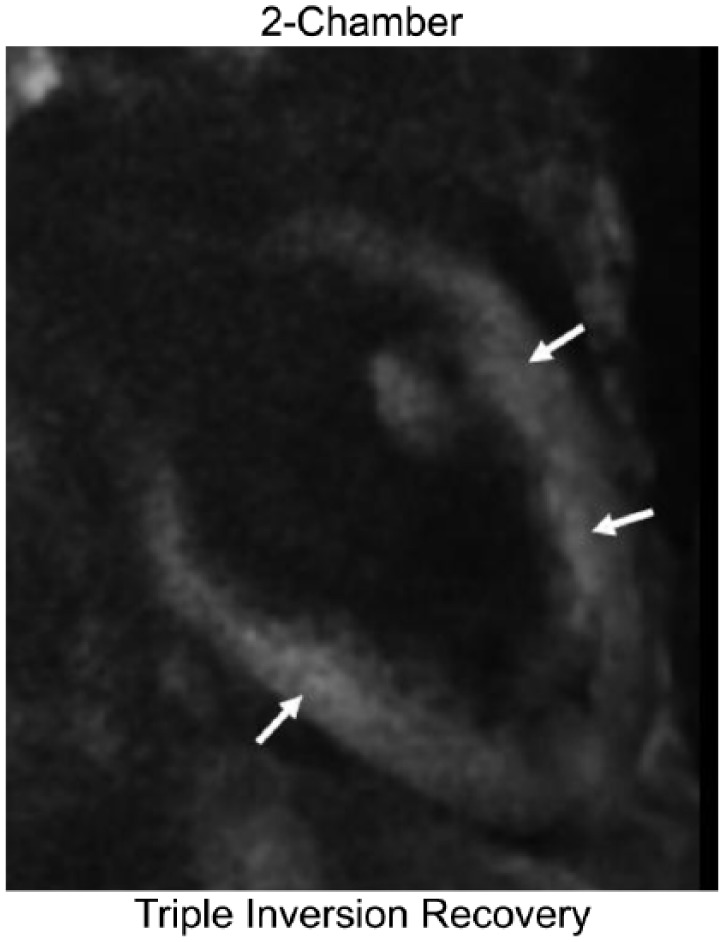
Cardiac magnetic resonance imaging triple-inversion recovery sequence demonstrating hyperenhancement consistent with myocardial edema (arrows).

A 48-year-old female with metastatic renal cell carcinoma, without any prior cardiac history or risk factors for cardiac disease, was admitted for scheduled high-dose IL-2 therapy. Prior to initiating her IL-2 regimen, the patient successfully completed 13 minutes of Bruce Protocol without any evidence of ischemia. She received 9 total doses over the course of 4 days (33 million IU every 8 hours), and therapy was discontinued because of significant diarrhea. On day 5, the patient complained of chest discomfort and was found to have an electrocardiogram (EKG) with ST segment depressions in the anterolateral leads (Figure 1) and troponin I level >50 ng/mL (reference <0.029). The patient subsequently developed acute respiratory failure and was intubated. Given the concerns for acute coronary syndrome, the patient was taken to the catheterization laboratory for coronary angiogram, which showed no evidence of obstructive coronary artery disease. Right heart catheterization showed elevated filling pressures bilaterally. Hemodynamic data included right atrial pressure 14 mm Hg, right ventricular systolic/diastolic pressures 52/17 mm Hg, pulmonary artery systolic/diastolic pressures 49/32 mm Hg, pulmonary capillary wedge pressure 26 mm Hg, and left ventricular systolic/diastolic pressures 97/37 mm Hg. Cardiac output and cardiac index, measured by thermodilution, were 7.2 L/min and 4.59 L/min/m^2^, respectively. Left ventriculogram showed global hypokinesis with ejection fraction (EF) of 35% and Seller grade II mitral regurgitation. An intra-aortic balloon pump was inserted for hemodynamic support and afterload reduction. Although her filling pressures were elevated, the patient had normal to high cardiac output with persistent systemic hypotension requiring vasopressor support suggestive of a component of distributive shock, a known side effect of IL-2. An echocardiogram also showed a reduced EF with moderate mitral regurgitation. Given the hemodynamic measurements consistent with distributive shock, the patient was initiated on multiple vasopressors. With hemodynamic support, the patient slowly improved and was able to be extubated, weaned off vasopressors, and the intra-aortic balloon pump was removed. However, on days 16 and 17 the patient had multiple episodes of sustained polymorphic ventricular tachycardia (PMVT) associated with hemodynamic instability requiring external defibrillations and amiodarone continuous infusion. On reviewing the initiation of her PMVT, patient was bradycardic and ventricular tachycardia (VT) was initiated by a single premature ventricular contraction (PVC) focus through R on T phenomenon (see Figure 2). A single atrial lead tempo-permanent pacemaker was placed, and programmed AAI 55 to prevent significant bradycardia, and the patient did not have any further episodes of PMVT. An endomyocardial biopsy was performed that showed fibrosis (Figure 3). A subsequent cardiac magnetic resonance imaging (MRI) was obtained once the patient no longer required pacing support. Left ventricular EF was quantified as 41%. There were significant areas of hypokinesis and scattered areas of subepicardial delayed gadolinium enhancement with thinning of the left ventricular wall and evidence of myocardial edema (Figures 4 and 5). Investigations for other causes of myocarditis were negative, including cytomegalovirus, influenza, coxsackie, aspergillus, mycobacteria, and blood cultures. The patient was discharged home on high-dose prednisone taper and given a defibrillator vest, with plans for repeat MRI in 3 months to assess need for an implantable cardioverter-defibrillator (ICD). A follow-up echocardiogram done 4 months after showed improved EF, and the patient was documented to have clinical improvement, and thus the decision to place an ICD was held.

## Discussion

The cardiac toxicities of IL-2 therapy have been well described in the literature. Myocarditis has been reported in 3% to 5% of patients receiving IL-2 therapy, and the myocardial dysfunction appears to be reversible.^3,4^ The pathophysiology of IL-2-induced myocarditis appears to be related to the release of tumor necrosis factor-α and cytokine-mediated myocardial insult, which also contribute to the development of a distributive shock type picture.^2,5,6^ Arrhythmias have been reported with IL-2 use; however, in the case reports most were atrial fibrillation and supraventricular tachycardias and resolved after discontinuation of therapy.^7,8^ Margolin et al studied 93 patients and showed one incidence of VT out of 20 cases of cardiac arrhythmias.^9^ Myocardial infarction can occur in patients with preexisting coronary artery disease; thus, cardiac assessment prior to treatment generally includes a baseline EKG and stress cardiac test in older patients or patients with family history of heart disease. We report a case of a patient who developed symptoms mimicking an acute coronary syndrome, with EKG changes, reduced left ventricular EF, and significantly elevated troponin levels with a normal coronary angiogram. An endomyocardial biopsy was not diagnostic, but a cardiac MRI showed findings consistent with myocarditis. One limitation of our case is that the biopsy was not conclusively diagnostic and that cardiac MRIs cannot definitively diagnose myocarditis. However, as our patient is a young female with no cardiac risk factors, absence of prior cardiac symptomatology, absence of coronary artery disease on angiogram, with acute elevation in cardiac enzymes, and evidence of edema on cardiac MRI, it is unlikely that her MRI findings were because of prior cardiomyopathy. Thus, the more likely scenario is myocarditis because of IL-2 therapy, although exacerbation of a preexisting cardiomyopathy cannot be completely ruled out without prior cardiac studies. Stress-mediated cardiomyopathy is also less likely given the delayed contrast enhancement on the MRI. Our case reiterates that noninfectious myocarditis can occur after IL-2 therapy and may present similarly to an acute myocardial infarction and timely diagnosis and discontinuation of therapy is important. This is rather unique because the patient developed a near-lethal ventricular arrhythmia, 11 days after IL-2 therapy was discontinued, and additionally required a temporary pacemaker with possibility of a permanent defibrillator given the severity of her cardiomyopathy.

We bring attention to the fact that although most cardiac toxicities from IL-2 appear to be reversible on discontinuation of therapy, lethal side effects that may potentially cause sudden cardiac death may develop days to weeks after the therapy cessation. It remains to be elucidated if higher incidence of arrhythmia is a dose-dependent phenomenon with use of IL-2 therapy similar to therapy with anthracyclines. Nonetheless, it is plausible that the underlying mechanism is because of myocarditis given that in vivo half-life of IL-2 is approximately 5 to 7 minutes, and our patient’s ventricular arrhythmia occurred more than 12 days after her last IL-2 infusion.^10^

Several rhythm disturbances have been observed in myocarditis, including sinus tachycardia, atrial, and, sometimes fatal, ventricular arrhythmias.^11,12^ Electrically sensitive foci in inflamed or scared areas because of myocarditis can give rise to PVCs. When a PVC occurs in a vulnerable period of a preceding T-wave, it can trigger ventricular arrhythmias, which is the likely mechanism responsible for VT in our patient. The exact underlying mechanism for developing ventricular arrhythmias in myocarditis, however, is not well understood. Additionally, release of cytokines and inflammatory mediators may affect ion channel function. Perhaps, acute myocarditis can also unveil preexisting channolopathies in prone individuals.^11^

Thus, cardiac evaluation prior to initiating treatment, including routine EKG and stress test, should be considered of great importance. There may be clinical value in obtaining a baseline echocardiogram as well to intensify screening of patients with any preexisting cardiac condition.
